# Making Sense of Oxidative Stress in Obstructive Sleep Apnea: Mediator or Distracter?

**DOI:** 10.3389/fneur.2012.00179

**Published:** 2012-12-27

**Authors:** Jing Zhang, Sigrid Veasey

**Affiliations:** ^1^Department of Pulmonary Medicine, Peking University First HospitalBeijing, China; ^2^Center for Sleep and Circadian Neurobiology, School of Medicine, University of PennsylvaniaPhiladelphia, PA, USA; ^3^Department of Medicine, School of Medicine, University of PennsylvaniaPhiladelphia, PA, USA

**Keywords:** neurons, obstructive sleep apnea, superoxide, redox regulation, carbonylation, cardiovascular diseases, intermittent hypoxia

## Abstract

Obstructive sleep apnea is increasingly recognized as an important contributor to cognitive impairment, metabolic derangements, and cardiovascular disease and mortality. Identifying the mechanisms by which this prevalent disorder influences health outcomes is now of utmost importance. As the prevalence of this disorder steadily increases, therapies are needed to prevent or reverse sleep apnea morbidities now more than ever before. Oxidative stress is implicated in cardiovascular morbidities of sleep apnea. What role oxidative stress plays in neural injury and cognitive impairments has been difficult to understand without readily accessible tissue to biopsy in persons with and without sleep apnea. An improved understanding of the role oxidative stress plays in neural injury in sleep apnea may be developed by integrating information gained examining neural tissue in animal models of sleep apnea with key features of redox biochemistry and clinical sleep apnea studies where extra-neuronal oxidative stress characterizations have been performed. Collectively, this information sets the stage for developing and testing novel therapeutic approaches to treat and prevent, not only central nervous system injury and dysfunction in sleep apnea, but also the cardiovascular and potentially metabolic conditions associated with this prevalent, disabling disorder.

## Introduction

Obstructive sleep apnea (OSA) syndromeis defined by the regular occurrence of episodic sleep state-dependent collapse of the upper airway resulting in disturbed ventilation and sleep. When ventilation ceases for greater than 10 s, the event is termed an apnea. When ventilation continues, but is reduced and associated with a significant decline in arterial oxygen saturation, the event is termed a hypopnea. Severity of the disorder is characterized by the frequency of apneas and hypopneas per hour of sleep; this measure is termed the apnea/hypopnea index. The cardinal features of the disorder, although not always present, are loud snoring with sporadic gasps in sleep associated with daytime sleepiness/fatigue or simply unrefreshed sleep. OSA is a common disorder in developed countries. Young et al. (1993) reported that approximately 4% of males and 2% of females have an apnea-hypopnea index greater than five events per hour of sleep combined with daytime excessive sleepiness. Using newer definitions for apneas and hypopneas, Duran et al. (2001) studied a 30–70 year old population-based sample and found an apnea/hypopnea index >15/h in 7% of women and 14% of males. There is a clear age-dependency for OSA in both females and males. In females the prevalence for OSA increases from 3% for the third decade of life to 36% in the seventh decade (Tufik et al., 2010). In men the prevalence for the third decade is 4% and increases to 50% for the seventh decade (Tufik et al., 2010). Although not all individuals with OSA are overweight, the vast majority of patients with OSA are obese (Millman et al., 1991). Obesity raises the prevalence in all groups. In children obesity increases the prevalence of OSA by over fourfold (Redline et al., 1999). This overlap of obesity and OSA in the majority of patients has made it more challenging to identify OSA vs. obesity contributions to cardiovascular and metabolic morbidities and is discussed below. Increased adiposity of obesity not only increases soft tissue volume within the upper airway, but also adds weight to the neck, further increasing airway collapsibility. In addition, central obesity reduces lung volumes which also promotes upper airway collapse. In addition to obesity, there are craniofacial features that predispose to upper airway collapse and OSA. For example both hypothyroidism and acromegaly result in increased upper airway soft tissue and OSA (Veasey, 2010). In addition, long-term allergic rhinitis, enlarged tonsillar-adenoid lymphoid tissue, nasal septal deformities, maxillary or mandibular insufficiency all impose increased collapsibility on the upper airway (Riha et al., 2009). Craniofacial syndromes with any of these features, for example Down’s, Apert’s, and Treacher Collins’ syndromes have increased prevalences of OSA (Katz et al., 2012).

The common underlying pathophysiology of OSA is a sleep state-dependent partial or complete collapse of the upper airway, resulting in brief interruptions in ventilation. In wakefulness, upper airway dilator muscles are activated to stent open the pharynx and hypopharynx in individuals with highly collapsible airways (Dempsey et al., 2010). Obstructive sleep-disordered breathing events occur because of normal sleep state-dependent reductions in activity, including the activity of upper airway dilators. Reduced dilator muscle tone combined with negative intraluminal pressures in the upper airway across inspiration result in upper airway collapse, with attendant hypoxemia and hypercapnia. The degree of hypoxemia relates directly to ventilatory responsiveness to both hypercapnia and hypoxia and also to end expiratory lung volume. Because of increased weight upon the abdomen and thorax, obese individuals will have smaller end expiratory lung volumes in sleep and therefore, more rapid declines in oxyhemoglobin desaturations. Both the hypoxemia and hypercapnia increase sympathetic output adding resistive load onto the heart. Attempts to inspire with an occluded upper airway result in large negative intrathoracic pressure swings, reducing cardiac preload, and increasing cardiac afterload. Coupled with the increased vascular resistance from the higher sympathetic drive cardiac output declines until the individuals arouses, restoring upper airway dilator muscle activity, airway patency, oxygen, and carbon dioxide levels. Each event then repeats a brief cycle of intermittent hypoxia, hypercapnia, sleep fragmentation, and arterial perfusion instability. What is unique to OSA is that these obstructive ventilatory disturbances occur only in sleep and individual events are quite brief, typically lasting less than 1 min. Of all of the above listed physiological perturbances, it is this frequently repeated hypoxia/reoxygenation that led investigators to examine the role of oxidative stress in OSA morbidities. This review provides the basic redox biochemistry necessary to accurately interpret the results obtained in previous OSA and oxidative stress studies and then integrates findings in animal models of sleep apnea oxygenation, where brain tissue has been examined with human studies where peripheral measures of redox have been analyzed.

## Biochemistry of Reactive Oxygen and Nitrogen Species

Upon first glance at the intricacies of pathways and seemingly countless molecules involved in oxidative modifications of proteins, lipoproteins, and lipids, renders understanding redox biochemistry a daunting task. But it need not be so. There are really just a few definitions to understand and several key endpoint oxidative modifications on the other end of the pathways to provide a knowledge base sufficient to understand oxidative injuries in OSA and interpret the seemingly contradictory results of previous studies. Reactive oxygen species (ROS) include superoxide $\left({\text{O}}_{2^{-}}∙\right),$ hydrogen peroxide (H_2_O_2_), hydroxyl radical (OH^−^•), hydroxyl ion (OH^−^), and hypochlorite ion (OCl^−^•). Not all ROS are free radicals; electrons are paired in both H_2_O_2_ and OH^−^. OH^−^• is the most reactive and can impart significant oxidative damage. There is a span of reactive nitrogen species ranging in low to high oxidized state from N_2_O to NO^−^ to NO to $\text{NO}+∕\text{N}{\text{O}}_{2^{-}}$ to N_2_O_3_ to NO_2_ to ONOO^−^ (peroxynitrite) to N_2_O_4_ to $\text{N}{\text{O}}_{3^{-}}$ (Gow, 2006).

## Healthy Oxidative Signaling

**Figure 1:**
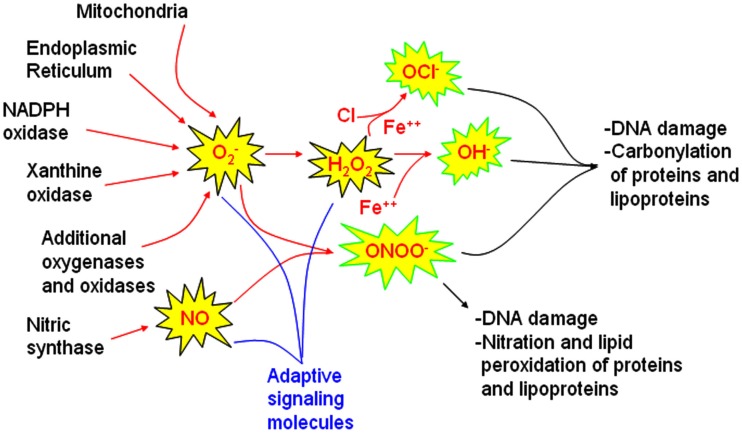
**Sources of reactive oxygen and nitrogen species involved in signaling and injury pathways**. Major sources of reactive oxygen species (ROS) identified in models of obstructive sleep apnea oxygenation include mitochondria, the endoplasmic reticulum, and NADPH oxidase. Whether xanthine oxidase and other oxidases or oxygenases also contributes to oxidative injury in sleep apnea oxygenation models requires further study. Nitric oxide synthases are also influenced by sleep apnea oxygenation patterns. These ROS and RNS sources may be activated for healthy signaling to increase superoxide $\left({\text{O}}_{2^{-}}∙\right),$ nitric oxide (NO) and hydrogen peroxide (H_2_O_2_). When these molecules are not rapidly cleared, hypochlorite (OCl^−^), hydroxyl (OH•) and peroxynitrite (ONOO^−^) radicals can develop. These highly reactive molecules are more likely to cause end-organ damage. Comprehensive examination of end-organ oxidative stress damage requires characterization of oxidative DNA damage, carbonyl, nitration, and lipid peroxidation measures.

To begin to understand oxidative stress, it is first important to recognize that certain ROS, e.g., ${\text{O}}_{2^{-}}∙$ and H_2_O_2_ and reactive nitrogen species, e.g., nitric oxide (NO) are important signaling molecules for adaptive, learning, and growth responses as illustrated in Figure 1. ${\text{O}}_{2^{-}}∙$ is essential for immunity, for killing specific bacteria and fungi. In the central nervous system, increased scavenging of ROS prevents hippocampal long-term potentiation that is necessary to consolidate spatial memory after learning trials (Klann, 1998). At the same time, too much ${\text{O}}_{2^{-}}∙,$ prevents long-term potentiation in the hippocampus (Gahtan et al., 1998). How can the same molecule be both adaptive and injurious? Reactive by nature, ${\text{O}}_{2^{-}},$ H_2_O_2_, and NO will have extremely localized effects, unless produced at excessive rates when these molecules may interact with a different subset of enzymes. For long-term potentiation, ${\text{O}}_{2^{-}}$ and H_2_O_2_ interact with calcium/calmodulin kinase II, protein kinase C, extracellular signal-related kinase, and calmodulin. When these ROS are produced at higher rates or are less consumed, they may inhibit calcineurin and inhibit long-term potentiation (Ferri et al., 2000). This is one mechanism by which both aging and Alzheimer’s may impair cognition (Agbas et al., 2005; Celsi et al., 2007). NO plays an important role in adaptive vasodilating responses in the peripheral vasculature and in neuronal signaling, where in both systems, NO serves to increase cyclic guanosine 3′,5′-(cyclic) phosphate (cGMP; Neo et al., 2010; Vincent, 2010).

## Cellular Sources of Oxidative Stress

### Mitochondria

In most cells, including neurons and glia, mitochondria are considered to be a major source of ROS. ${\text{O}}_{2^{-}}∙$ is a normal byproduct in ATP production at complexes I and III along the electron transport chain. In fact 1–4% of oxygen consumed will be converted into ${\text{O}}_{2^{-}}∙.$ Under healthy conditions, the ${\text{O}}_{2^{-}}∙$ is converted to H_2_O_2_ by SOD2 and then to H_2_O by glutathione peroxidase in the mitochondria or by catalase if it enters the cytoplasm. Mitochondrial redox homeostasis is very tightly regulated, where mechanisms of mitochondrial redox regulation are only now being discovered. In mammals, seated right at complex I is sirtuin 3 (SirT3). This NAD^+^-dependent deacetylase is activated by increased energy need and serves to activate complex I (Ahn et al., 2008) and at the same time SirT3 initiates an impressive antioxidant response, (Bell and Guarente, 2011). SirT3 increases reduced glutathione by activating both isocitrate dehydrogenase 2 and glutathione dehydrogenase (Someya et al., 2010; Yu et al., 2012). For added mitochondrial protection, SirT3 activates Fox03a to increase transcription of both superoxide dismutase isoform 2 (SOD2) and catalase and directly activates SOD2 (Qiu et al., 2010). The adaptive SirT3 response diminishes with advanced aging and contributes to age-dependent hearing loss. Indeed, the ability of caloric restriction to reduce oxidative stress and promote survival in auditory neurons is completely dependent on SirT3 (Someya et al., 2010). Thus, SirT3 is an example of an antioxidant regulator that targets not just one component of the antioxidant response but numerous components. Understanding the molecules with far reaching effects on redox responses will provide more effective therapies against oxidative damage. As mentioned above Complex III also contributes to the formation of ROS and under hypoxic conditions, where ubiquinone reduction products are major sources of ROS. Finally, ROS may be produced in the mitochondria outside the electron transport chain, within the Krebs cycle by an enzyme, alpha-ketoglutarate dehydrogenase (Tretter and Adam-Vizi, 2004). Whether Sirt3 directly influences these latter two sources of ROS will be important to discern in future studies, but it is also important to understand that SirT3 transcription itself may be inhibited by high amounts of ROS (D’Aquila et al., 2012).

### Endoplasmic reticulum

One of the most oxidative environments in the cell is within the lumen of the endoplasmic reticulum (ER), where the highly oxidative environment is necessary for the formation of specific disulfide bonds in protein folding, essential for a protein’s activity. In health, disulfide formation is tightly regulated by protein disulfide isomerases, and is prevented from randomly occurring in this highly oxidative environment by chaperones. The primary functions of the ER are to process and fold proteins. Pathophysiological conditions, such as OSA, diabetes, aging, and obesity, can lead to increased misfolded proteins in the ER, overwhelming the availability of chaperones, thereby causing further protein misfolding with improper disulfide bonds (Malhotra et al., 2008). A vicious cycle may be set in these conditions as oxidative modifications in proteins from the underlying disease result in protein misfolding and then require greater ER re-folding activities and thus greater oxidative stress. Severe ER oxidative stress results in calcium release into the cytoplasm, that, in turn, increases oxidative stress in the mitochondria through many of the mechanisms listed above in mitochondrial sources of ROS. Increased ROS in the ER also depletes reduced glutathione further compounding oxidative stress. This ER and mitochondrial oxidative stress crosstalk sets up a second vicious cycle where oxidative stress in one organelle facilitates the development in a second.

### ROS and RNS generating enzymes

Within many eukaryotic cells, including neurons and glia there are numerous oxidases and oxygenases that can contribute to increased bioavailability of ROS and RNS. One of the most prominent is NADPH oxidase (Nox). NADPH oxidase directly increases ${\text{O}}_{2^{-}}∙$ and may play a central role in activating other sources of ${\text{O}}_{2^{-}}∙,$ including mitochondria (via opening mitochondrial transition pore), xanthine oxidase, but activating xanthine oxidase and RNS by uncoupling both endothelial nitric oxide synthase (eNOS) and neuronal nitric oxide synthase (nNOS; Ago et al., 2011). Nox2 is prominent in the brain and can be activated by angiotensin II, protein kinase C activation, and by increased intracellular calcium. Herein lies another vicious cycle in that increased intracellular calcium will activate both mitochondrial and NADPH oxidase sources of ${\text{O}}_{2^{-}}∙,$ resulting in more ER stress and more intracellular calcium. eNOS activation can sequester ${\text{O}}_{2^{-}}∙$ through production of ONOO^−^ and is believed to play a largely protective role until oxidative stress suppresses its activity via oxidizing its co-factor BH_4_.

## Considerations of Redox Assays

One of the many challenges in oxidative stress research is the vast number of possible assays available with which to characterize oxidative stress. This can be simplified by considering what the different assays measure. In general the assays fall into several groups: RNA and DNA damage, lipid peroxidation, protein nitration, protein oxidation, and the presence of ROS. Some of the more commonly used ROS assays for nucleic acid modifications include 8-hydroxyguanosine (8-OHG) and 8-hydroxydeoxyguanosine (8-OHdG), both of which can be measured in tissue, blood, and urine. The gold standard for lipid peroxidation is mass spectroscopy for 8-isoprostanes, but 4-hydroxynonenal (4-HNE), malondialdehyde (MDA), and thiobarbituric acid reactive substances (TBARS) are also used. 4-HNE cannot be sampled in urine, while others can. Protein oxidation is measured as total protein carbonyl by ELISA or westerns, and 3-nitrotyrosine is used to characterize nitration of proteins, also by westerns and ELISA. Dihydroethidine allows histological examination of superoxide availability, as described below in animal studies (Nguyen et al., 2003). There are many newer *in vivo* fluorescent reporters for ROS that will allow subcellular localization of mitochondria or ER ROS production. Additionally, examination of NO availability can be used as an index of superoxide availability. One of the newer techniques being optimized for more direct measurement of ROS, RNS, and oxygen is electron paramagnetic resonance (EPR) spectroscopy (Mrakic-Sposta et al., 2012). EPR may be used for plasma, tissue culture, and even *in vivo* experiments. The technique is sensitive enough to detect exercised-induced ROS in the plasma of healthy adults, and values correlate very closely with TBARS and carbonyl protein levels (Mrakic-Sposta et al., 2012).

There are several important considerations when selecting oxidative stress measures to characterize OSA redox. Examination of total proteins will include long-lasting proteins with cumulative redox changes and short-lived proteins with most recent changes. In sleep apnea, it would be helpful to identify proteins with very rapid turnover to allow measures before and after sleep or before and after a night of therapy. Conversely, examination of long-lasting proteins allows a long-term snapshot of oxidative status. It is hoped that future studies will parcel out for analysis several short-lived proteins susceptible to nitration and/or oxidation changes and several proteins susceptible to long-term cumulative oxidative and nitrative changes.

## Evidence for Neuronal Oxidative Stress in Animal Models of OSA

### Redox alterations associated with neuronal injury or dysfunction

Although many of the physiological perturbations described in the above Introduction have the potential to increase the bioavailability of ROS and RNS, the best-studied challenge in animal models is intermittent hypoxia, modeling the patterns of severe OSA arterial oxygenation. It is important to recognize that mild, brief episodes of intermittent hypoxia may actually be good for the brain. Mitchell and colleagues have shown that exposure to <1 h of mild intermittent hypoxia increases long-term facilitation of the phrenic and hypoglossal nerves and promotes increased growth after spinal cord injury (Baker and Mitchell, 2000; Lovett-Barr et al., 2012). Long-lasting more frequent episodes, however, clearly result in neuronal injury and cognitive dysfunction (Gozal et al., 2001). There are areas of the brain that are more susceptible to this long-term intermittent hypoxia (LTIH) injury, namely pyramidal neurons in the cerebral and hippocampal cortices, Purkinje neurons, catecholaminergic wake-active neurons, and motoneurons, particularly trigeminal, facial, and hypoglossal motoneurons (Gozal et al., 2001; Veasey et al., 2004a,b; Pae et al., 2005). Carbonylated proteins, tyrosine nitration, and lipid peroxidation are all found in the brains of animals exposed to LTIH (Veasey et al., 2004a,b). In each of these groups it appears that superoxide contributes to injury or neuronal dysfunction because superoxide dismutase mimetics administered throughout the LTIH exposure can prevent or substantially reduce injury or dysfunction (Peng and Prabhakar, 2003; Veasey et al., 2004b). A newer technique applied by Douglas et al. (2010) allows a look at superoxide availability. Injected systemically, dihyroethidine is readily taken up in all cell bodies and nuclei. In the presence of superoxide radical, dihydroethidine converts to autofluorescent dihydroethidium/ethidium that may be analyzed by fluorescent microscopy. Douglas et al. (2010) found increased superoxide radical availability in cortical neurons of newborn mice exposed to intermittent hypoxia.

### NADPH oxidase neural injury in LTIH

The differential in vulnerability across groups of wake neurons led to the discovery that NADPH oxidase subtype 2 (Nox2) is a major source of oxidative injury (Zhan et al., 2005). Since this initial discovery, NADPH oxidase has been shown to contribute to LTIH hippocampal and cortical injury (Yuan et al., 2008; Nair et al., 2011). Of great clinical significance, NADPH oxidase is also implicated in many LTIH vascular injuries or conditions, including hypertension, cardiac remodeling, and pulmonary hypertension (Hayashi et al., 2008; Khan et al., 2011). Thus, NADPH oxidase may be an excellent pharmacological target for preventing and treating neuronal and vascular injuries secondary to intermittent hypoxia in OSA.

### Mitochondrial injury in LTIH

In addition to NADPH oxidase injuries, mitochondria are injured in model of LTIH. Mitochondria are a source of ROS in *in vitro* models of LTIH (Peng and Prabhakar, 2003). As the mitochondrial isoform of superoxide dismutase (SOD2) prevents cortical neuronal injury (Shan et al., 2007), mitochondria must also be an important source of ROS in LTIH. Douglas et al. (2010) have shown that mitochondrial dysfunction precedes oxidative stress in a model of LTIH with intermittent hypercapnia. The source of mitochondrial dysfunction in LTIH is not known. Whether mitochondrial injury results in NADPH oxidase activation or vice versa will be important to examine to identify the optimal targets for antioxidant therapies in OSA.

### ER stress injury in LTIH

Our lab has recently examined the role of ER stress in LTIH motoneuron injury, finding that the unfolded protein response is activated in hypoglossal and facial motoneurons by LTIH and that this results in severe ER stress with upregulation of the C/EBP homologous binding protein (CHOP; Zhu et al., 2008). In LTIH CHOP increases oxidative stress by upregulating an ER oxidase (ERO-1), hypoxia-inducible factor-1a, and NADPH oxidase (Nox2; Chou et al., in press). Whether CHOP influences mitochondrial release of ROS should now be examined.

### Xanthine oxidase in ischemic injury

Xanthine oxidase is an important source of oxidative stress in reperfusion injuries to the brain and heart. Whether xanthine oxidase inhibitors are effective in reducing neuronal injury in LTIH has not been reported. Xanthine oxidase inhibition does not always prevent or modify ischemic injury (Arai et al., 1998) and can however have detrimental effects including lowering NADH in the brain (Al-Gonaiah et al., 2009). Peripherally, there is evidence that xanthine oxidase contributes to endothelial dysfunction in LTIH and then administration of allopurinol, a xanthine oxidase inhibitor, can reduce LTIH oxidative stress (Williams et al., 2010; Dopp et al., 2011).

In summary, oxidative stress is clearly an important contributor to neuronal injury in LTIH animal models of OSA oxygenation. Whether the other physiological perturbations including hemodynamic changes, sleep fragmentation, and carbon dioxide fluctuations also modify ROS production should be examined. In LTIH, NADPH oxidase, mitochondria, and ER stress are all involved in ROS generation. Reducing one source does influence other sources, but the optimal combination still eludes us and must be confirmed to improve mitochondrial function. As we move forward to advance therapeutics to reduce neuronal injury in OSA, we will need to consider the role of ROS in cardiovascular morbidities as well.

## Peripheral Oxidative Stress in Human OSA

### Evidence for lipid peroxidation in OSA

There have been many clinical studies examining lipid peroxidation in OSA. Some of the challenges in these studies small sample sizes (inadequate statistical power) and include subjects with confounding co-morbidities, e.g., obesity and cigarette smoking, that were not always matched across groups (Barcelo et al., 2000; Ozturk et al., 2003). Lavie et al. (2004) examined TBARS in 114 patients with OSA and cardiovascular disease and compared levels to 55 patients with cardiovascular disease but without OSA. TBARS in OSA, relative to non-OSA, subjects was twice as high before and after sleep, suggesting a sustained increase in lipid peroxidation from OSA without an acute increase from one night of untreated OSA (Lavie et al., 2004). A positive correlation was found for the severity of OSA and the TBARS value (Lavie et al., 2004). Across a collection of subjects with moderate-severe OSA, plasma MDA levels decrease with continuous positive airways pressure (CPAP), but remain well above normal values (Jordan et al., 2006). Svatikova et al. (2005) measured plasma TBARS and isoprostanes in moderate-sever OSA subjects before and after 4 h of therapy for OSA with CPAP and found no treatment effect. This duration of therapy is not expected to affect total lipid peroxidation. In a trial looking at MDA plasma levels in severe OSA patients before and after 10 months of therapy, MDA levels fell significantly (Hernandez et al., 2006). An alternative therapy for mild OSA is an oral mandibular advancement device that moves the lower jaw forward; this therapy has also been shown to reduce MDA levels (Itzhaki et al., 2007). Overall, it seems that OSA contributes to elevated lipid peroxidation in individuals with moderate-severe OSA. The absence of compete normalization of MDA levels with effective therapy for OSA over sufficient time to replace many cells contributing to plasma MDA levels, supports the concept that the higher MDA in patients with OSA (but without pulmonary, cardiac, or endocrine disorders) is in part a consequence of co-morbid conditions, including obesity, high fatty diet, sedentary lifestyle.

### Evidence for protein nitration in OSA

Several studies have examined protein nitration also in plasma. As with lipid peroxidation measures, in many studies details are not provided as to how rapidly plasma was dissociated and plasma frozen. This is a problem as substantial nitration and lipid peroxidation may continue in unfrozen blood, plasma, and urine samples. A recent study reported both lipid peroxidation and protein nitration levels in both exhaled breath condensates and plasma for 35 patients with moderate-severe OSA (AHI > 15/h) before and after 3 months therapy with CPAP. Exhaled breath condensate nitration fell significantly from 17 to 5 pg/ml, *p* < 0.05, as did 8-isoprostane levels, 6–3, *p* < 0.05. Larger changes were observed for plasma values. In contrast c-reactive protein, interleukin-6 and tumor necrosis factor-a did not change significantly in this group of patients with therapy (Karamanli et al., 2012). Jelic and colleagues performed endothelial cell biopsies on individuals with mild, moderate, and severe OSA and controls and then used immunohistochemistry techniques on rapidly fixed cells to measure nitration, eNOS, and iNOS. Subjects with OSA had higher levels of nitrotyrosine and cyclooxygenase-2 (COX2) and lower levels of eNOS. With CPAP therapy, nitrotyrosine and COX2 levels normalized. This work strongly supports the concept that OSA can increase nitration in cells, exhalate, and plasma (Jelic et al., 2008). That therapy fully reverses nitration suggests that OSA itself is a primary contributor to protein nitration.

### Evidence for increased protein carbonylation and superoxide in OSA

Protein carbonylation occurs hyperbolically across aging, but at a great pace with prolonged oxidative stress. There have been several studies examining protein carbonylation in OSA patients. In one of the first carbonylation was examined in intercostals muscle tissue (Barreiro et al., 2007). Consecutive patients were examined in a clinic population without matching for health problems in controls. Carbonyl levels were higher in OSA subjects; however, values did not change with 6 months of effective CPAP therapy. Thus, it is not clear whether OSA contributes to protein carbonylation in skeletal muscle. A second study reported serum carbonyl levels across the spectrum of OSA patients findings a positive correlation between AHI and serum carbonyl levels (Vatansever et al., 2011). There is one study reporting improvements in plasma carbonyl levels with 3 months of CPAP therapy (Jurado-Gamez et al., 2011). It is important to recognize that many structural proteins in the brain exist for a lifetime and thus may accumulate carbonyl modifications over years, leading to progressive misfolding, impaired function, highlighting the importance of early intervention. There is one pilot EPR study examining the effect of leptin administration on superoxide in plasma of OSA patients. The study shows a dose-dependent response for leptin in lowering ROS (Macrea et al., 2012). The study lacks control groups for comparison of basal ROS, but shows the promise of using this technique to evaluate treatment effects on ROS in individuals with OSA.

## Circadian Considerations of OSA and Oxidative Stress

A large body of research over the past few years has unveiled important, albeit complex, relationships between the circadian system, metabolics, and OSA. The prevalence of metabolic syndrome is >10% in adults with OSA, and when patients with both OSA and metabolic syndrome (without other co-morbidities) are treated effectively with CPAP, the metabolic derangements reverse sufficiently to lower the prevalence of metabolic syndrome to 1% in treated individuals (Sharma et al., 2011). At the cellular level metabolics is primarily influenced by redox changes in nicotinamide adenine dinucleotide (NAD+) and flavin adenine dinucleotide (FAD). Recently, one of the major enzymes involved in NAD+ synthesis, NAMPT, was found to demonstrate a strong circadian rhythm (Nakahata et al., 2009; Ramsey et al., 2009). NAD+ influences the activity of SirT1 and SirT3 and, thus, is expected to contribute to circadian rhythms of antioxidant enzymes. In simpler life forms many antioxidant enzymes demonstrate circadian rhythmicity (Yoshida et al., 2011). One of these antioxidant enzymes, peroxiredoxin, shows circadian rhythmicity also in mice (Edgar et al., 2012). Intriguingly, peroxiredoxin levels in the liver and SCN of the mouse cycle 180° out of phase (Edgar et al., 2012). Thus, we must consider patients’ circadian rhythmicity and antioxidant rhythmicity within specific tissues as key variables in assessing redox status in individuals with OSA, before and after therapy.

## Conclusion and Prospective

As the prevalence of obesity escalates worldwide, so does the prevalence of OSA, including in children and young adults. In children, OSA is linked to poor performance in school (Beebe and Byars, 2011). In adults with OSA, impaired cognition is associated with increase risk of motor vehicle accidents and poor work performance (Shah et al., 2006). Many studies examining cognitive performance and brain imaging studies in OSA show improvements with therapy but incomplete resolution (Li and Veasey, 2012). Studies in animal models of OSA oxygenation patterns support oxidative injury as a major contributor to neuronal injury in OSA, where NADPH oxidase activation and mitochondrial and ER dysfunction underlie the surge in ROS. Providing a non-invasive whole organ or plasma measurement of ROS, further refinement of EPR will provide a much-needed window into oxidative stress in OSA and in animal models of the disorder. In humans, such measures may be used to guide clinical therapeutic options in patients and to follow effectiveness of therapy. ER and mitochondrial stress and HIF-1a upregulation clearly underlie, at least in part, the oxidative stress. For example ER stress protein CHOP is necessary for NADPH oxidation, ROS, and HIF-1a activation and should be an excellent upstream target for the prevention of oxidative injury in OSA (Yuan et al., 2008). It is clear from human OSA studies that similar mechanisms are at play in the periphery. Thus, the periphery may provide a valuable window into the severity of OSA oxidative stress in individual patients, potentially allowing therapeutic individualization. For the numerous children and adults with OSA, these steps must be taken expeditiously to minimize irreversible injury.

## Conflict of Interest Statement

The authors declare that the research was conducted in the absence of any commercial or financial relationships that could be construed as a potential conflict of interest.
